# Attention control in preterm and term 5-month-old infants: Cross-task stability increases with gestational age

**DOI:** 10.1111/infa.12574

**Published:** 2024-01-20

**Authors:** Oliver Perra, Alice Winstanley, Rebecca Sperotto, Merideth Gattis

**Affiliations:** 1 School of Nursing and Midwifery, Queen's University Belfast, Belfast, UK; 2 School of Psychology, Cardiff University, Cardiff, UK

## Abstract

Cross-task stability refers to performance consistency across different settings and measures of the same construct. Cross-task stability can help us understand developmental processes, including how risks such as preterm birth affect outcomes. We investigated cross-task stability of attention control in 32 preterm and 39 term infants. All infants had the same chronological age at time of testing (5 months) but varied in gestational age (GA) at birth (30–42 weeks). Infants completed an experimental attention following task with a researcher and a naturalistic play observation with their mothers. Both preterm and term infants demonstrated attention following in the experimental task. GA and flexibility of attention were related: the likelihood of no turn trials decreased with increasing GA. To evaluate cross-task stability, we compared attention performance in the experimental and naturalistic settings. Flexible attention shifts on the experimental task were positively related to attention to objects in the naturalistic observation. Furthermore, the association between flexible attention shifts on the experimental task and attention to objects in the naturalistic observation was moderated by GA. Our study provides initial evidence that the consolidation of attention control increases with GA. These findings highlight the value of comparing experimental and observational measures of attention.

## 1 Introduction

Visual attention plays a foundational role in the development of cognitive and communicative skills (Çetinçelik et al., 2021; Salley et al., 2016; Tenenbaum et al., 2015). For example, visual fixation and tracking during early infancy are positively associated with cognitive performance on standardized assessments at 5 years (Stjerna et al., 2015). Furthermore, preterm birth, defined as birth before 37 weeks gestation, is associated with impairments to both visual attention during infancy and cognition during childhood and adulthood (Burstein et al., 2021; Ginnell et al., 2021; Kaul et al., 2022). Importantly, however, current evidence indicates that at least some attention skills are intact in preterm infants (Burstein et al., 2021; van de Weijer-Bergsma et al., 2008). We evaluated cross-task stability in visual attention, defined as performance consistency across different settings and measures, in healthy preterm and term infants. Infants were 5 months from birth when they completed attention assessments, but they varied in gestational age (GA) at birth. We tested infants' attention orienting and control in an experimental task and compared their performance on the experimental task to performance in naturalistic interactions with their mothers. In introducing this study, we first provide an overview of attention processes in infancy. We next focus on evidence concerning visual attention in preterm infants. We then highlight the need to investigate attention across different settings and measures to evaluate cross-task stability.

### 1.1 Visual attention in infancy

A key milestone in the development of attention is when infants become able to orient and control attention flexibly (Hendry et al., 2016; Posner & Rothbart, 2007; Ruff & Rothbart, 1996). Visual attention is less flexible in newborns compared to older infants, in part because very young infants have difficulty shifting attention from one stimulus to another (Johnson & De Haan, 2015). For example, Hood and Atkinson (1993) showed that 3-month-olds, but not 2-month-olds, reliably disengaged attention from a centrally located image of a face and oriented toward a peripheral stimulus. Most researchers argue that disengagement is initially controlled exogenously, or in other words, it is driven by environmental stimulation (Atkinson & Braddick, 2012). Nonetheless, disengagement is important because it is a first step toward endogenous attention control, that is the ability to direct attention flexibly and voluntarily to gather information (Hendry et al., 2016).

Endogenous attention control is frequently indexed by controlled experimental measures of sustained attention or novelty preference (Burstein et al., 2021; Colombo & Cheatham, 2006). As endogenous attentional control develops, infants begin to flexibly shift attention between stimuli, such as a person and object, thus setting the stage for sharing attention with social partners (Salley & Colombo, 2016). Attention following is therefore another useful approach for evaluating endogenous attention control. By 6 months, most infants begin to follow attention when a social partner shifts their eyes or head to look at an object, if the object is within the infant's field of view (Del Bianco et al., 2019). For example, D'Entremont et al. (1997) evaluated attention following in 3-to 6-month-olds by coding infants' gaze shifts in response to an experimenter who held two puppets, one near each shoulder, and alternated looking at the infant with looking at either puppet. Infants made more correct turns (to the same side as the experimenter) than incorrect turns or no turns. Attention following emerges at around 3 months in healthy, typically developing infants born at term and is negatively associated with autism symptoms at 18 months (Perra & Gattis, 2010; Thorup et al., 2018).

### 1.2 Visual attention in preterm infants

Preterm infants have difficulties orienting and controlling attention (Burstein et al., 2021; Downes et al., 2018; Gattis, 2019). For example, Rose et al. (2001) presented preterm and term infants with a familiar stimulus beside a novel stimulus and coded attention shifts between the two. To control for differences in physical maturity, Rose et al. (2001) matched preterm and term infants on age since conception, thus adjusting for GA at birth. This matching procedure is known as testing infants at their *corrected age*. At 5, 7, and 12 months corrected age, preterm infants displayed fewer attention shifts between stimulus pairs. Rose and colleagues interpreted this difference between preterm and term infants as evidence of a deficit in endogenous attention control in infants born preterm, even when controlling for differences in physical maturity.

Current accounts of how attention develops do not clearly delineate when and how preterm birth influences attention, and importantly, at least some attention skills are not negatively impacted by preterm birth (Burstein et al., 2021; Gattis, 2019; van de Weijer-Bergsma et al., 2008). One reason for the lack of clarity is the challenge of defining appropriate comparisons between preterm and term infants. In studies using corrected age, preterm and term infants are matched on physical maturity but differ in extrauterine experience, and importantly, experience with visual stimuli influences attention orienting and control (Butcher et al., 2002; Hunnius et al., 2008). Peña et al. (2014) compared preterm and term infants in a computerized attention following task. Preterm infants in the study were 4- and 7-months corrected age, and therefore had approximately 3 months additional extrauterine experience compared to term infants of the same maturity, or days since conception. Extrauterine experience mattered: at 4 months corrected age, preterm infants followed attention more frequently than 4-month-old term infants. Furthermore, preterm infants of 4 months corrected age followed attention just as frequently as 7-month-old term infants. In other words, preterm and term infants with equivalent extrauterine experience performed similarly, even though they differed in birth status and in physical maturity.

A clear account of when and how preterm birth influences attention needs to consider evidence from naturalistic as well as experimental measures. Gattis et al. (2020) evaluated orienting and responding to attention in preterm and term infants during naturalistic interactions with their mothers. To control for extrauterine experience, Gattis et al. (2020) matched preterm and term infants on chronological age: all infants were observed 5 months after they were born. Importantly, infants' physical maturity varied depending on their GA at birth, which ranged between 30 and 42 weeks. The researchers evaluated the frequency and duration of infant attention to objects and persons as well as responding to attentional bids. Infants of lower GA spent less time focused on objects compared to more mature infants and were less responsive to their mothers' attention bids to objects. Because infants in their study differed in GA but had the same amount of extrauterine experience, and were otherwise healthy, Gattis et al. (2020) proposed that physical maturity influences the development of flexible attention control. In the study presented here we build on Gattis et al. (2020) by comparing infant attention in the naturalistic observations they described with performance in an experimental attention following task. In the next section we argue that cross-task stability can help researchers evaluate how attention develops, including how preterm birth affects development.

### 1.3 Cross-task stability of visual attention

One motivation for researchers to assess infant attention experimentally is to evaluate and describe the underlying processes: experimental manipulations allow stronger inferences about what allows or causes attention (e.g., D'Entremont et al., 1997; Hood & Atkinson, 1993). A corresponding limitation, however, is that experiments are necessarily narrow: experiments evaluate a particular attentional behavior in a particular context. Abelkop and Frick (2003) measured duration of looking amongst 4- and 6-month-olds during a non-social, perceptual task and a controlled social interaction with the mother, the still face paradigm. Infants who looked at non-social perceptual stimuli for briefer durations showed greater differences in looking across the three phases of the still face paradigm: they looked at their mothers longer during their initial interaction, less during the still face, and more during the reunion. Based on this pattern of performance across the two different settings, Abelkop and Frick (2003) proposed that the attentional processes measured in experimental perceptual tasks also affect infant behavior in social domains. They noted, however, that the stability of look duration across contexts may vary, both with age and task demands.

Although cross-task stability has a long history in developmental psychology, surprisingly few studies have evaluated the cross-task stability of visual attention during infancy (Roberts & Pomerantz, 2004; Vaughn et al., 1984). Furthermore, studies that have evaluated the cross-task stability of visual attention during infancy have produced limited evidence of performance consistency across tasks (e.g., Rose et al., 2004; Striano et al., 2009; Wass, 2014). In one longitudinal study, Perra and Gattis (2010, 2012) evaluated infant attention in experimental and naturalistic settings on a monthly basis from two to 4 months. In the experimental task, based on the paradigm developed by D'Entremont et al. (1997), an experimenter held two identical puppets, one near each of his shoulders, and engaged the infant before turning to look at one of the puppets. The experimenter repeated this behavior for multiple trials in a predetermined order. Infants' attention shifts to the same puppet as the experimenter (correct turns) were compared against shifts to the other puppet (incorrect turns) and continuing to look at the experimenter (no turns). At the group level, infants first demonstrated more correct turns than incorrect and no turns at 3 months. Correct turns were followed by continued shifts of attention between target and experimenter, or *checking back*, which also emerged at 3 months. In all the testing sessions, infants and parents completed a naturalistic play observation in an adjacent room prior to the experimental task. Perra and Gattis (2012) evaluated infant attention in the naturalistic observations using Bakeman and Adamson's (1984) coding system for describing states of engagement. Infants who spent more time attending to objects held by their parent at 2 months displayed more checking back in the attention following task at 3 months (Spearman's *ρ* = 0.33, *p* < 0.05). Furthermore, infants who displayed similar or increasing proportions of time attending to objects held by their parent at 3 and 4 months displayed more attention shifts between target and experimenter at 4 months. Perra and Gattis (2012) thus demonstrated cross-task stability of infant visual attention.

Very few studies have investigated cross-task stability of attention in preterm infants. One exception is de Jong et al. (2015), who evaluated attention abilities in preterm and term infants at 18 months corrected age using eye tracking, observations, and parent report. Infants' on-task persistence during target-directed interactions with the caregiver (reading a book and solving a puzzle) was significantly and positively correlated with experimental assessments of attention using eye-tracking, in particular attention alerting (*r* = 0.21, *p* < 0.01) and attention orienting (*r* = 0.23, *p* < 0.01). Further studies of cross-task stability are needed, particularly in younger infants. Furthermore, comparisons of preterm and term infants' performance across tasks can inform researchers and practitioners about the impact of preterm birth on the development of attention.

### 1.4 The current study

Our overarching goal was to evaluate attention in preterm and term infants, including their performance on a controlled experimental task, the extent to which performance on the experimental task was consistent with performance during naturalistic observations (cross-task stability), and relations with GA. These analyses build on earlier results from naturalistic observations of the same preterm and term infants at the same age (Gattis et al., 2020). The experimental task, based on D'Entremont et al. (1997) and Perra and Gattis (2010), allowed us to evaluate flexible control of attention, as indexed by attention following and checking back in response to an experimenter's turn to look at a target within the infant's visual field. Comparisons between the experimental task and the naturalistic observations allowed us to evaluate cross-task stability of attention. We observed infants at 5 months based on evidence that endogenous attention control first emerges between 3 and 6 months (Del Bianco et al., 2019; Hendry et al., 2016; Salley & Colombo, 2016). Infants were matched on chronological age to ensure that all infants had the same length of extrauterine experience (5 months from birth). Infants varied in GA at birth and our analyses considered GA both categorically (preterm infants vs. term infants) and continuously (30–42 weeks GA). To control for co-morbidities as potential confounders, the sample included only healthy term and preterm infants. The aims of this study were to evaluate:

1. Infant attention on the experimental task, including attention following, checking back, and no turns, and relations between those variables and GA;
2. Cross-task stability, defined here as relations between attention on the experimental task and attention in the naturalistic observations; and
3. Relations between cross-task stability and GA, as a test of the hypothesis that the consolidation of attention control increases with GA.

## 2 Method

### 2.1 Participants

All recruitment and testing procedures were reviewed and approved by the Cardiff and Southeast Wales Local Research Ethics Committee and were consistent with the ethical principles outlined in the Declaration of Helsinki. All participants received study information when their child was born and provided written informed consent to participate before any assessment or data collection. A total of *N* = 71 participants (30 females: 42%) completed at least one valid session of the experimental task (see Section 2.2) and were thus retained in the analyses. We report socio-demographic characteristics of preterm and term infants in Table 1. Preterm infants differed in GA and birth weight but did not differ in sex or maternal education (Table 1). All assessments took place in a single visit to a university lab when infants were 5 months (±15 days) and therefore the chronological age of preterm and term infants was similar by design (Table 1).

The 71 participants included in this study because they completed the attention following task represented 68% of the *N* = 104 that completed the naturalistic observations described in Gattis et al. (2020). Infants with complete data did not differ significantly in sex and maternal education from the 104 included in Gattis et al. (2020). However, they differed in GA: infants who completed the attention following task displayed increased odds of being preterm (OR = 2.56, 95% CI 1.02–6.46) and their GA was 1.56 weeks younger than those with missing data (95% CI −2.83 to −0.30). Thus, non-completion of the experimental task was associated with maturity, rather than immaturity. In Supplementary Information S4 and S5 we replicated key analyses using multiple imputation to obtain reliable estimates of performance across infants with partial missing data, thus testing our models on the 104 who participated in the previous study. Data are available on request from the corresponding author.

### 2.2 Design and procedures

The overall study used a cross-sectional, correlational design in which infants completed two attention assessments. One assessment used an experimental procedure, while the other assessment involved naturalistic observations of infants and their mothers.

**Figure 1:**
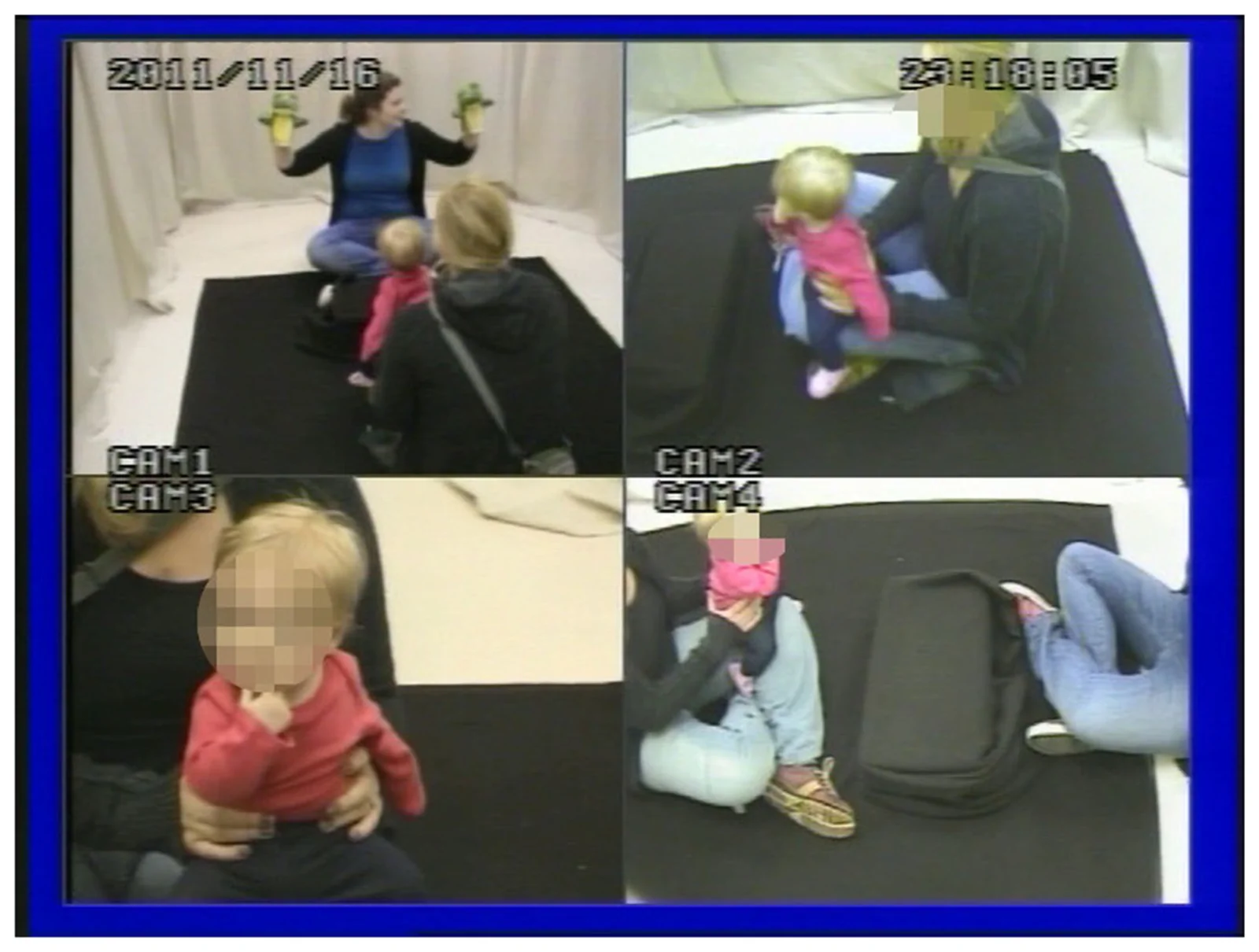
Example of videorecording output of the experimental task. The infant's and mother's face have been pixelated to ensure anonymity.

All assessments of infant attention took place on the same day in a quiet, infant-friendly university laboratory. Assessments were videorecorded by four cameras placed at different angles of the room. Camera outputs fed into a quad video-mixer, thus allowing synchronous recording of all outputs, with some cameras focusing on the infant's face and others focusing on the experimenter or the mother. An example of the video-recording output is displayed in Figure 1. One output (Cam 1 in Figure 1) focused on the experimenter, and the experimenter was often visible in a further output (Cam 4 in Figure 1). The other outputs (Cam 2 and Cam 3 in the Figure 1) focused on the infant and the experimenter was not visible.

#### 2.2.1 Experimental attention following task

Infants sat on their caregiver's lap while the caregiver sat on the floor (see Figure 1). A female experimenter sat approximately 40 cm from the infant and caregiver while wearing identical glove puppets on her two hands and held them at shoulder height. The experimenter attracted the infant's attention by talking to the infant in infant-directed speech: when she judged the infant was looking at her, she turned her head and eyes toward one of the two puppets, and kept this posture for 7 s while continuing to talk in infant-directed speech. An attention following trial was defined as the 7 s period where the experimenter turned her head to one puppet and remained oriented to it. After the first trial, the experimenter resumed eye contact with the infant before repeating the procedure three more times for a total of four trials. The directions of the experimenter's head turns were Right-Left-Left-Right or Left-Right-Right-Left, with the two orders counterbalanced across infants.

A coder blind to infant birth status coded all trials from the videorecords using Interact software (Mangold International, 2016). Coding was carried out in two runs. In the first run, the coder identified the start and end of each trial and coded: (1) the direction of the experimenter's turn (left vs. right); (2) the distance between the infant and the experimenter (40 cm vs. more than 40 cm); (3) whether the head of the infant was supported by the mother (yes vs. no); (4) the infant's state (drowsy, calm, mild protest, distress). In the second run, the same researcher coded infants' gaze shifts throughout each 7 s trial, based on the start and end times identified in the first run. Paper covered the portion of the screen where the experimenter's head appeared (*Cam 1* and *Cam 4* output in Figure 1) to ensure the researcher was blind to the experimenter's direction of head turn. The researcher coded infants' gaze shifts into mutually exclusive categories: Ahead (infant looking ahead at the experimenter's head); Right (infant gaze directed to the right horizontal plane); Left (infant gaze directed to the left horizontal plane); Away (infant gaze directed in any other direction, e.g., looking down). Additional categories were used to indicate if the infant's gaze was not visible (e.g., infant moving outside the camera frame) or if the infant's eyes were closed continuously for 1 s or more. These additional categories ensured exhaustive coding of infants' behavior.

A second coder, also blind to infants' birth status and the experimenter's direction of head turn, coded all the sessions of 24 infants (one-third of all the infants who provided data). We used Cohen's *κ* to calculate agreement with a strict time tolerance for mismatches of 1 s. All *κ*s were satisfactory: distance between the infant and the experimenter *κ* = 1.00 (100% agreement); whether the infant's head was supported by the mother *κ* = 0.71 (91% agreement); infant's state *κ* = 0.90 (98% agreement); experimenter's direction of turn *κ* = 1 (100% agreement); and infants' gaze direction *κ* = 0.80 (86% agreement).

A valid attention following trial was defined as a trial in which the infant looked ahead, that is, directly at the experimenter's face, when the experimenter's head turn started; trials where the infant was not looking ahead when the experimenter's head turn started (e.g., infant looking to one side) were excluded from analyses. Trials where the infant's gaze was not visible at the start of the trial were also excluded. Overall, 60 (21%) of all trials administered (*N* = 288) were considered invalid. A negative binomial regression indicated that the number of completed trials was not significantly associated with GA (IRR = 1.02, *p* = 0.32). Preterm infants completed 3.03 trials on average (SD = 1.03) while term infants completed 3.36 trials on average (SD = 0.84), but this difference was not significant, *t*(69) = 1.47, *p* = 0.14.

#### 2.2.2 Naturalistic observations

The methods and results for the naturalistic observations were reported by Gattis et al. (2020). For convenience we summarize the procedure and coding from Gattis et al. (2020) below. A researcher provided three toy bins that contained 15 age-appropriate toys and asked the caregiver to play with their child “as you would normally do at home.” To facilitate videorecording, mothers were asked to ensure the infant was positioned on a rectangular mat (130/190 cm) at the center of the room (see Figure 1). Four cameras in the room (Figure 1) obtained synchronous recordings of the infant and the mother from four different angles; this ensured that the faces of both infant and mother, as well as objects being manipulated, were visible in the recording. The observations lasted 15 min for all dyads, except three that terminated at 13 min: data from these observations were prorated to 15 min.

Infant attention was coded in three mutually exclusive and exhaustive categories as (1) looking at mother, (2) looking at an object, or (3) neither of the above. In the current study we focus on one specific index of infant attention from Gattis et al. (2020), the proportion of the interaction that infants spent in object-oriented attention, here called *Attention to Objects*. This measure was obtained by summing the duration of all infant looks to objects and calculating it as a proportion of the 15 min observations. To correct for an asymmetric distribution of this variable, the raw proportions were transformed by calculating their square.

Maternal attention was coded in mutually exclusive and exhaustive categories as: (a) encouraging attention to herself, (b) encouraging attention to an object, or (c) neither of the above. Sequential analysis (Bakeman & Quera, 2011) was then used to create variables that described behavioral streams. In the current study we focus on one specific sequence from Gattis et al. (2020) whereby infants looked at an object when the mother was encouraging attention to the same object, here called *Responding to Attention*. This code was only applied if the infant response occurred within 3 s of the onset of the initial behavior. For each dyad, time units were tallied in 2 (Infant looking at object *or* other) by 2 (Mother encouraging attention to object *or* other) tables; these tables enabled the calculation of the odds ratios (ORs) of infants' responding to their mother's object-directed attention by looking at the same object (see Gattis et al., 2020). In data analyses these ORs were transformed using their natural logarithm to ensure ease of interpretation and symmetrical distributions. Log-odds equal to 0 indicate no difference in the odds of an infant responding by looking at the object or not. Negative log-odds indicate the infant is less likely to respond by looking at the object. Positive log-odds indicate the infant is more likely to respond by looking at the object.

One coder had coded infants' behavior, while a second one coded mothers' behavior, thus ensuring independent event coding. The roles of the two coders were reversed for reliability coding that involved 20% of the interactions: agreement was calculated on a second-to-second basis. The agreement obtained was *κ* = 0.66 for maternal behavior and *κ* = 0.58 for infants' behavior, which was deemed acceptable (Gattis et al., 2020).

## 3 Results

The analytic plans and results are organized below by the three study aims. All analyses were conducted using Stata 17.0 (StataCorp, 2021).

### 3.1 Aim A. Infant attention on the experimental task

#### 3.1.1 Plan of analyses

Based on the coding (described in Section 2.2), we assigned each valid trial in the attention following task to one of four mutually exclusive and exhaustive response categories. The response categories were: *Attention Following* if the infant's first gaze turn was in the correct direction, that is, the same direction as the experimenter; *Incorrect Turn* if the infant's first gaze turn was in the incorrect direction, that is, the direction opposite to the experimenter; *Look Away* if the first infant's gaze turn was in a direction other than to left or right; *No Turn* if the infant did not shift direction of gaze. All Attention Following trials were further coded to evaluate whether the infant subsequently looked back to the experimenter, a behavior dubbed *Checking Back*. A trial was categorized as Checking Back if an infant showed the following sequence of gaze shifts from the start of the trial: Experimenter → Target → Experimenter.

For each infant we calculated the proportion of Attention Following, Checking Back, and No Turn trials using the number of valid trials as the denominator. On Attention Following trials the infant correctly followed the experimenter's attention and looked at the same target. Checking Back trials were only possible for Attention Following trials. On No Turn trials the infant looked only at the experimenter in front of them, so neither Attention Following nor Checking Back was possible. Preliminary analyses indicated that these proportions were not affected by contextual factors within trials, that is, the distance between the infant and experimenter and whether the infant's head was supported by the mother (Supplementary Information S1). The Look Away code ensured that the assignment of responses to categories was exhaustive but was not readily linked to specific attention processes and we therefore did no further analyses for Look Away responses.

To test if preterm and term infants followed attention, we evaluated whether the proportion of Attention Following trials was significantly higher than that expected by chance. Because Attention Following trials were one of four possible response categories, we set chance level as *p* = 0.25. We then tested if preterm and term infants displayed different proportions of Attention Following, Checking Back, and No Turn trials using two-sample Wilcoxon rank-sum tests.

To test whether GA was associated with proportion of Attention Following, Checking Back, and No Turn trials, we regressed these outcomes on GA (centered at 37 weeks), testing both linear and quadratic associations. Since the proportions of Attention Following, No Turn, and Checking Back were calculated on four trials at most, the range of scores displayed was limited and the distributions were not normal: to account for this, we ran ordinal logistic regressions whereby the outcome of interest (e.g., proportion of No Turn trials) was considered an ordered categorial response. The main parameter of interest is a coefficient that represents how the probability of higher outcome scores changed for each additional week of gestation (see Supplementary Information S2 for more details).

#### 3.1.2 Aim A results

Infants completed an average of 3.2 valid trials (range 1–4). Table 2 displays the average proportions of the four response types by birth status. Overall, 80% (*n* = 57) of infants displayed Attention Following in at least one trial. Preterm infants displayed a higher proportion of Attention Following trials than expected by chance, *t*(31) = 3.83, *p* = 0.001, as did term infants, *t*(38) = 3.40, *p* = 0.002. Preterm and term infants did not display a significant difference in their proportion of Attention Following trials, Wilcoxon rank sum test *z* = −0.72, *p* = 0.48.

Preterm and term infants displayed similar proportions of Checking Back trials. The average proportion of Checking Back trials was 0.26 (SD = 0.27) for preterm infants, and 0.27 (SD = 0.26) for term ones; these proportions were significantly different from zero in preterm (*t*[31] = 5.49, *p* < 0.001) and in term infants (*t*[38] = 6.52, *p* < 0.001). The difference in Checking Back trials between preterm and term infants was not significant, Wilcoxon rank sum test *z* = 0.22, *p* = 0.82.

Preterm and term infants differed in their proportions of No Turn trials, that is, trials where the infant looked only at the experimenter for the whole 7 s trial. Overall, 34 infants (48%) displayed at least one No Turn trial and a higher percentage of these infants, *n* = 19 (56%), were preterm. In addition, preterm infants displayed a higher proportion of No Turn trials compared to term infants, 0.30 and 0.18, respectively (Table 2). A Wilcoxon rank sum test confirmed this difference between preterm and term infants was significant, *z* = −1.85, *p* = 0.032 (unidirectional hypothesis).

**Figure 2:**
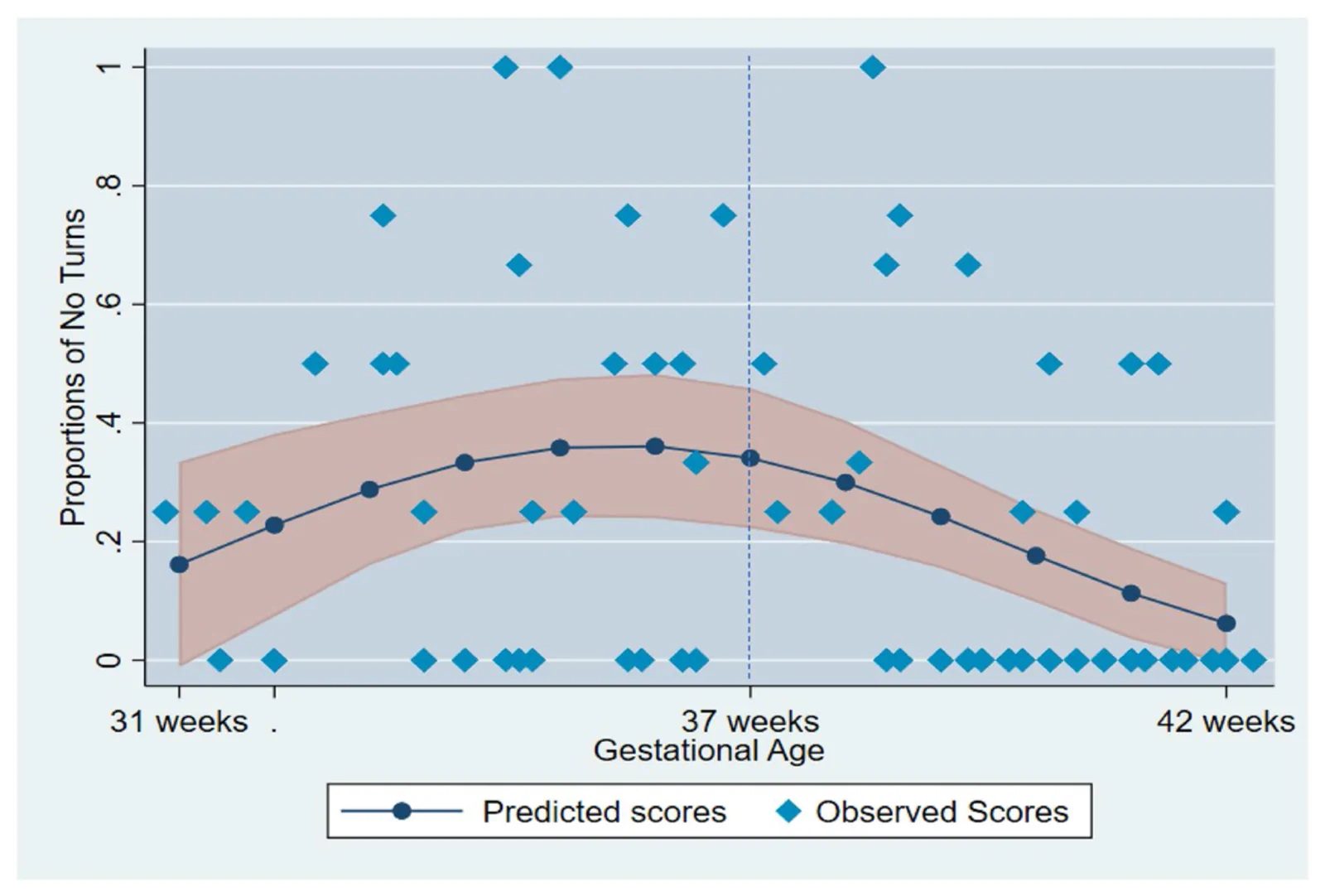
Proportions of No Turn responses in the attention following task by gestational age (GA). The light blue diamonds represent observed scores. The dark blue dots and the continuous line that unites them represent the scores predicted according to the quadratic trend. The shaded area represents 95% Confidence Intervals of predicted scores. We also report the conventional term age (37 weeks GA). The predicted scores reported in this graph were based on a multilevel logistic model that considered No Turn trials (level 1) nested within infants (level 2).

Further analyses tested the linear and quadratic trends of these outcomes in association with infants' GA. Infants' Attention Following trials were not linearly associated with GA (*χ*^2^[1] = 0.42, *p* = 0.52), nor did Attention Following trials display a quadratic trend across GA (*χ*^2^[2] = 2.48, *p* = 0.29), see Supplementary Information S2. Ordinal logistic regressions also did not indicate significant associations between proportions of Checking Back trials and GA, whether considering a linear trend (*χ*^2^[1] = 0.08, *p* = 0.78) or a quadratic one (*χ*^2^[2] = 0.14, *p* = 0.93), see Supplementary Information S2. Conversely, the initial regression indicated a marginal linear change in the proportion of No Turn trials across GA, *χ*^2^(1) = 3.60, *p* = 0.058. A further regression indicated that the proportion of No Turn trials followed a quadratic trend across GA, *χ*^2^(2) = 8.70, *p* = 0.013. The latter model provided a significant increase in model fit compared to the initial linear trend model, *Likelihood Ratio Test χ*^2^(1) = 5.10, *p* = 0.024. The parameters of this regression indicated that while the likelihood of higher proportions of No Turn decreased with GA, *coefficient* = −0.18 (95% CI −0.33 to −0.03), the probability of higher proportions of No Turn trials decreased more steeply with increasing GA, *coefficient* = −0.05 (95% CI −0.10 to −0.01). We represent this trend in Figure 2.

In subsequent analyses we focused on the proportion of Checking Back as the main outcome from the attention following task, since it indicates infants' flexible control of attention. Checking Back trials necessarily entailed Attention Following and were negatively and significantly correlated with No Turn trials (Spearman's *ρ*[69] = −0.29, *p* = 0.015).

Supplementary Information S4 reports additional analyses demonstrating that the same pattern of results was replicated for Attention Following, No Turns, and Checking Back when including all 104 participants who took part in the original study. These analyses used multiple imputation of missing values in the experimental outcomes.

### 3.2 Aim B. Relations between the experimental task and naturalistic observation

#### 3.2.1 Plan of analyses

To evaluate relations between attention on the experimental task and attention in the naturalistic observations, we regressed two infant outcomes from the naturalistic observations (Attention to Objects and Responding to Attention, reported in Gattis et al. (2020)) on the proportion of Checking Back trials. In these regressions we controlled for GA (centered at 37 weeks) by including it as a covariate. We used a robust estimator of variance to allow for non-normality of the distribution of Checking Back proportions (Supplementary Information S3).

#### 3.2.2 Aim B results

As previously reported in Gattis et al. (2020), Attention to Objects and Responding to Attention were positively associated with GA, *r*(69) = 0.25, *p* = 0.037 and *r*(69) = 0.39, *p* = 0.001 respectively. Attention to Objects correlated with the proportion of Checking Back trials, Spearman's *ρ*(69) = 0.24, *p* = 0.046.

In contrast the correlation between Responding to Attention and Checking Back was small and not significant, Spearman's *ρ*(69) = −0.002, *p* = 0.99. Regressions controlling for GA (Table 3) confirmed that Checking Back was positively and significantly associated with Attention to Objects, *β* = 0.19, *p* = 0.047. Checking Back was not associated with Responding to Attention, *β* = −0.05, *p* = 0.67.

### 3.3 Aim C. Relations between cross-task stability and gestational age

#### 3.3.1 Plan of analyses

To evaluate relations between cross-task stability and GA we ran a regression model whereby GA (centered at 37 weeks) moderated the association between the variables from the experimental and naturalistic settings. To this end, we created an interaction term multiplying GA by the proportion of Checking Back trials (Supplementary Information S3). The interaction term allowed us to test whether the association between Attention to Objects and Checking Back varied depending on GA (see Hayes (2017) for more details about moderation models). In running this regression, we used a robust estimator of variance to allow for the non-normal distribution of Checking Back. We compared the moderation model to the nested model tested in the previous section (Aim B) using the percentage of outcome variance explained by each model (*R*^2^).

#### 3.3.2 Aim C results

**Figure 3:**
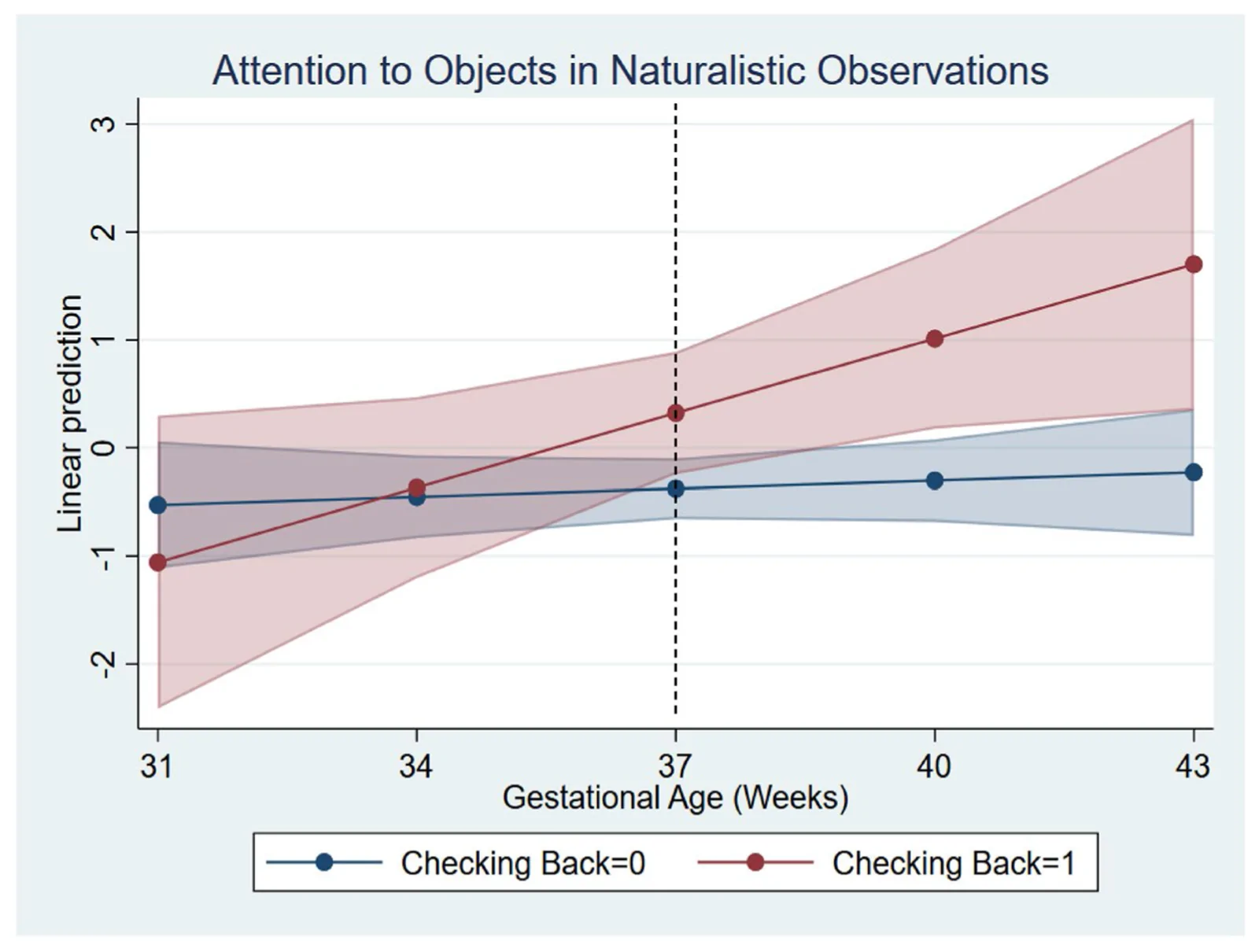
Predicted scores for attention to objects during naturalistic observations as a function of gestational age (GA) and proportion of Checking Back trials. The two lines represent the two ends of the possible range of scores in the proportion of Checking Back trials, where the blue line indicates no Checking Back in any trial (score = 0) and the red line indicates Checking Back displayed in every trial (score = 1). These two points were chosen for illustrative purposes. Shaded areas represent 95% Confidence Intervals. We indicate the conventional term age (37 weeks GA).

The moderation model demonstrated a good fit to the data, *F*(3,67) = 3.44, *p* = 0.022, Root MSE = 0.916. Model parameters are reported in Table 4. The interaction term was marginally significant, *β* = 0.24, *p* = 0.09. The moderation model explained a higher percentage of variance in the outcome, *R*^2^ = 0.13, compared to the nested model, *R*^2^ = 0.10. Figure 3 illustrates that the association between the two tasks was moderated by GA.

To further test whether cross-task stability differed by GA, we considered each infant's rank in Attention to Objects (naturalistic observations) and Checking Back (experimental task). We allowed for ties by giving the same rank to infants with the same score (e.g., two infants who displayed Checking Back in all the attention following trials obtained rank 1). For each infant we then calculated the difference between ranks on the two tasks, following a procedure similar to the one used in calculating Spearman's correlation coefficient. We reasoned that if cross-task stability increased with GA, the absolute difference in the ranks of the two measures would decrease with increasing GA. We therefore expected to find a significant negative correlation between differences in ranked scores and GA.

**Figure 4:**
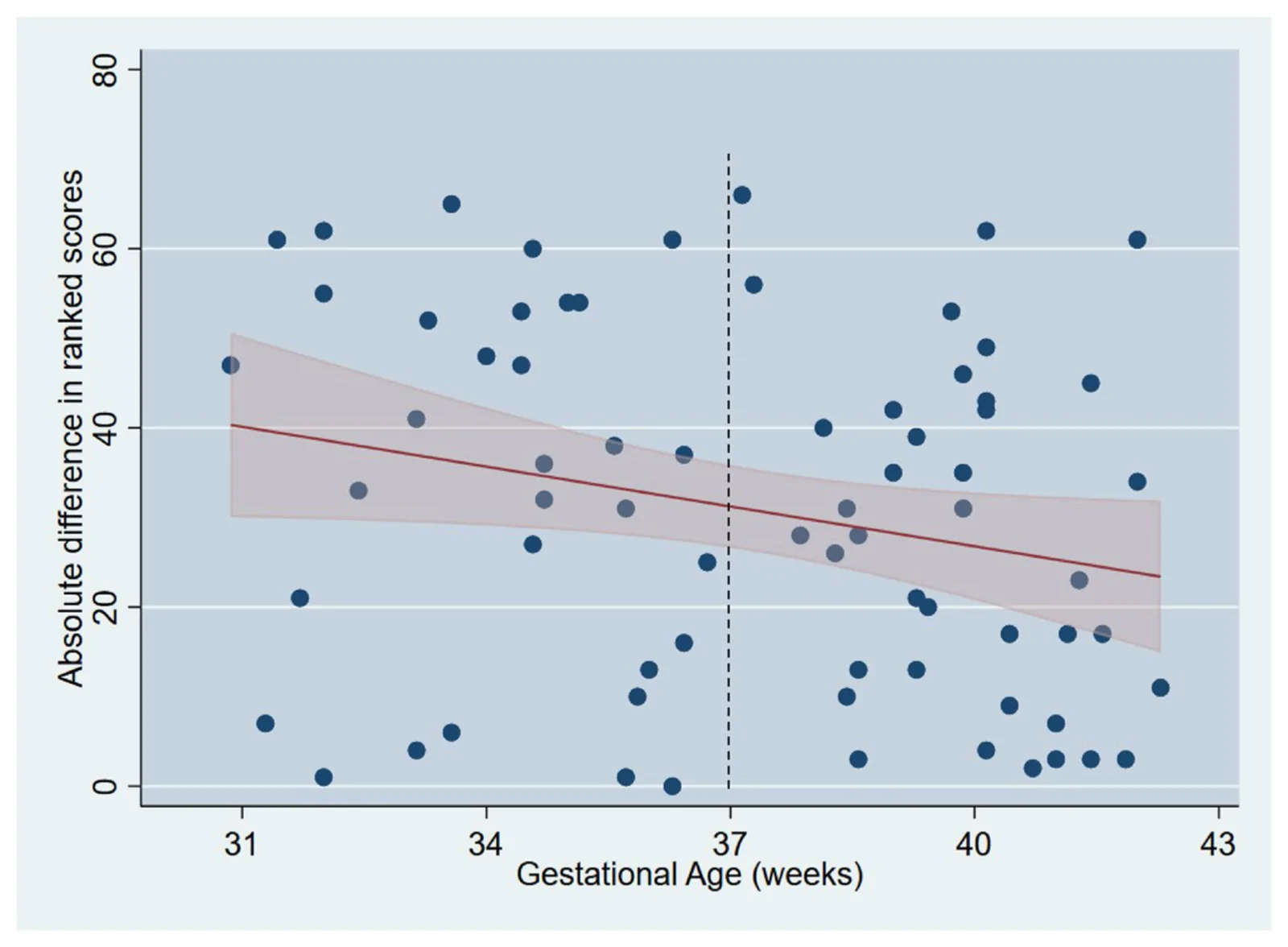
Scatterplot and linear trend of infants' absolute differences of ranked scores in attention to objects (naturalistic task) and checking back (experimental task) by gestational age (GA). The blue dots represent observed scores. The red line represents the predicted linear trend. The shaded area represents the 95% Confidence Intervals of the linear trend. The dashed vertical line indicates the conventional term age (37 weeks GA).

The results confirmed a significant negative correlation between absolute differences in ranked scores and GA, Spearman's *ρ*(69) = −0.24, *p* = 0.040. A regression confirmed the linear association between these variables was significant and negative *β* = −0.24, *t* = −2.07, *p* = 0.042 (*F*[1,69] = 4.28, *p* = 0.042, Root MSE = 19.49, for the whole equation). Absolute differences in ranked scores across the two tasks decreased linearly according with infants' GA, as indicated in Figure 4. Differences in ranked scores did not show a quadratic trend across GA, *F*(2,68) = 2.27, *p* = 0.112, Root MSE = 19.59. The pattern of results reported in this section were consistently replicated in analyses run on the *N* = 104 participants with complete and incomplete data (Supplementary Information S5). These analyses were run using multiple imputation methods (Supplementary Information S5).

## 4 Discussion

This study evaluated attention in preterm and term infants by assessing performance in a controlled experimental task and comparing performance on the experimental task with attention in naturalistic observations, thus testing cross-task stability. We also assessed relations between cross-task stability and GA. Preterm and term infants had the same chronological age and therefore the same length of extrauterine experience but differed in physical maturity because their GA ranged from 30 to 42 weeks at birth. We evaluated attention at 5 months, a transitional age when flexible attention control is emerging (Del Bianco et al., 2019; Hendry et al., 2016; Salley & Colombo, 2016).

Our first aim was to evaluate attention following in an experimental task. Both preterm and term infants followed attention by turning in the same direction as the experimenter. Preterm and term infants also displayed the same rate of checking back, defined as shifting attention between target and experimenter, and thus demonstrated flexible attention control (Perra & Gattis, 2010). Preterm infants differed from term infants in disengagement: more preterm infants had at least one trial during which they remained looking at the experimenter for the entire trial and such failures to disengage were negatively related to GA. Our study thus revealed a nuanced picture of strengths and difficulties in infants born preterm: although both preterm and term infants demonstrated attention following and checking back, infants of lower GA had some difficulties with endogenous attention control, as indicated by differences in disengagement. Our results build on existing studies of infant attention following in controlled settings and contribute further evidence that infants begin to share attention with social partners during early infancy (Del Bianco et al., 2019; Gredeback et al., 2010; Mendoza-Garcia & Moreno-Nunez, 2023). In addition, our results confirm evidence that preterm and term infants with equivalent extrauterine experience perform similarly on at least some measures of attention, even when they differ in birth status and physical maturity (Peña et al., 2014).

Our second aim was to evaluate consistency of attention performance in the experimental and naturalistic settings, or cross-task stability. We focused on checking back as the key indicator of flexible attention control in the experimental setting and our analyses controlled for GA (Perra & Gattis, 2010, 2012). Flexible attention control in the experimental setting was positively related to sustained attention to objects in the naturalistic setting but unrelated to responding to attention. Our results are consistent with those of Perra and Gattis (2012), who reported that attention control in healthy infants born at term was related across experimental and naturalistic settings at 3 and 4 months, and with their proposal that sharing attention to objects with social partners during early infancy relies on emerging attention control. The pattern of relations across settings may indicate that naturalistic observations are a more demanding context for infant attention: although responding to attention bids in the naturalistic setting might seem equivalent to attention control in the experimental setting, those behaviors were unrelated, whereas sustained attention to objects in the naturalistic setting was related to attention control. One possibility is that naturalistic settings require more effortful control due to greater variability of contextual factors, including speech and other noises as well as other types of physical stimulation. If so, naturalistic settings may be a more robust indicator of attention abilities.

Our third aim was to test relations between cross-task stability and GA. The association between flexible attention control on the experimental task and attention to objects in the naturalistic observation was moderated by GA, such that infants of greater GA displayed stronger associations between checking back in the experimental task and attention to objects during the naturalistic observations. In addition, differences in performance across the two settings decreased with GA, thus indicating increasing stability across the two tasks. To our knowledge our study is the first to evaluate the relations between cross-task stability of attention and GA. de Jong et al. (2015) reported moderate cross-task stability of attention in preterm and term infants at 18 months corrected age but did not test whether cross-task stability was associated with GA. Our results demonstrate the value of considering how GA, and relatedly physical maturity, might influence not only performance on a single task, but the stability of performance across tasks.

### 4.1 Cross-task stability of visual attention in infancy

Studies evaluating cross-task stability have the potential to inform theoretical accounts of development. More specifically, although behavior varies across tasks and situations, numerous studies have demonstrated that cross-task stability increases with age and emergent stability is taken as an indicator of the consolidation of some underlying capacity that yields greater performance consistency (e.g., McCall, 1981; Roberts & Pomerantz, 2004; Vaughn et al., 1984). Evidence of infants' performance consistency across different measures of visual attention is limited (e.g., Rose et al., 2004; Striano et al., 2009; Wass, 2014). Our study indicates a moderate degree of stability in preterm and term infants' flexible attention control across different task settings at 5 months. Furthermore, cross-task stability was moderated by GA, such that the relation between attention behaviors across tasks was stronger for infants of greater GA, consistent with the claim that the consolidation of attention control increases with maturity. All infants in our study had the same chronological age so that we could control for extrauterine experience, but infants varied in GA at birth and therefore physical maturity. The observed relation between cross-task stability and GA in our study is consistent with the results of other studies demonstrating that cross-task stability varies with physical maturity. Although we controlled for comorbidities during participant recruitment, we must also consider the possibility that developmental abnormalities associated with preterm birth other than maturity per se might have contributed to this observed pattern.

An open question remains about the extent to which experimental versus naturalistic measures are more accurate indicators of emergent abilities, including attention in early infancy. Some researchers advocate for controlled experimental tasks as a more accurate indicator of developmental abilities because they minimize and control for contextual variability (Wass & Smith, 2014). Long-term predictive relations between infant attention in experimental tasks and later cognitive and meta-cognitive abilities during childhood and adolescence accord with this view (Bornstein et al., 2013; Cuevas & Bell, 2014; Stjerna et al., 2015). Other researchers argue that less controlled observations capture life as it is lived by identifying emergent abilities in the context of naturally occurring variability and longer observations (Ellis-Davies et al., 2012; Repetti et al., 2015). Studies of infant attention in naturalistic settings have also yielded evidence of long-term predictive associations with later cognitive attainments, thus supporting this argument (Brandes-Aitken et al., 2019; Johansson et al., 2016). For example, Lawson and Ruff (2004) observed preterm infants at 7 months corrected age during free play with toys and reported that focused attention in the naturalistic setting predicted standardized cognitive assessments at 2, 3, and 4 years. Overall, the results of both types of studies emphasize the value of assessing infant attention across different settings to help researchers evaluate the functionality and consolidation of attention skills and to inform theoretical accounts of how such skills develop.

Following Abelkop and Frick (2003), we have used the phrase cross-task stability to describe performance consistency across different settings and measures of the same construct, even when measured at the same age. Stability refers to consistency in the relative order or rank of individuals whose behavior is assessed on different occasions and may be most informative when those occasions are distributed across longer periods of time, such as months or years (e.g., Bornstein et al., 2013, 2017). Our study assessed consistency of performance on two tasks completed on the same day, providing valuable information on consistency of attention abilities across settings in a narrow time frame during early infancy. Evaluations of cross-task stability in attention over extended developmental periods are needed to help researchers further evaluate the continuity, stability, and consolidation of attention skills.

### 4.2 Preterm birth and attention development

Our study contributes further evidence that the relations between preterm birth and attention skills are diverse and nuanced (Burstein, et al., 2021; Downes et al., 2018). In the experimental setting, preterm infants demonstrated above-chance levels of attention following and checking back, but consistent with Rose et al. (2001), did have some difficulties with disengagement. Our results point toward the importance of evaluating attention in a range of settings as well as considering how both experience and maturity contribute to attention development. Preterm and term infants in our study had the same length of visual experience and performed similarly on attention following and checking back, consistent with the proposal of Peña et al. (2014) that experience has a stronger influence than maturity on the development of these attention behaviors. We note however that Gattis et al. (2020) observed an effect of maturity on attention orienting for the same infants in a naturalistic setting, which may be a more challenging context for attention control. Furthermore, the analyses of cross-task stability reported here reinforce the view that maturity influences performance consistency. Peña et al. (2014) compared preterm infants with two groups of term infants, one group matched on experience and the other group matched on maturity. Such dual-matching designs, combined with assessments of flexible attention control in different settings, will be useful in future studies to better evaluate the relative contributions of experience and maturation on attention development.

Gattis (2019) contrasted two theoretical accounts of how preterm birth influences child health and development. According to the maturation perspective, preterm birth creates a period of developmental vulnerability, but with appropriate care and maturation, differences between preterm and term infants diminish. According to the maturation perspective, the emergence and consolidation of flexible attention control requires longer in preterm infants because they need additional time to mature but these differences should decrease with age. In contrast, the divergence perspective emphasizes the sensitivity of developmental processes to environmental inputs, particularly in terms of the timing of inputs, and argues that deviations in timing lead to increasingly different developmental trajectories for preterm and term infants. According to the divergence perspective, the differences in flexible attention control we and Gattis et al. (2020) observed between preterm and term infants at 5 months will increase at later ages and additionally have consequences for other aspects of development such as communication and language. Longitudinal evidence is thus critical for a fuller understanding of how preterm birth impacts on the development of attention and related skills.

### 4.3 Limitations and future directions

Our study used robust methods and reliable, fine-grained coding systems to evaluate the stability of attention control in infants with different GAs. Our results highlight the value of combining controlled and naturalistic assessments of attention to inform theories of how attention abilities develop, particularly following preterm birth. Our findings are limited, however, by their cross-sectional nature. Future studies should compare attention performance across settings longitudinally to evaluate the developmental consequences of preterm birth. As noted above, studies of attention following preterm birth should also consider dual-matching designs to better evaluate the contributions of experience and maturation to attention development.

Compared to previous studies that used a similar attention following paradigm (D'Entremont et al., 1997; Perra & Gattis, 2010), infants in our sample displayed lower proportions of attention following and higher proportions of other responses. In addition, term infants were less likely to complete the attention following task compared to preterm infants. Together these results suggest that infants in our study, particularly the term infants, were less engaged in the attention following task compared to previous studies with younger infants and to the preterm infants in this study. Although we cannot be certain, we tentatively propose that the attention following task, which has a slow pace and limited stimulation, may be more appropriate for younger infants, as in Perra and Gattis (2010). We tested infants at 5 months based on previous evidence that the task was suitable for 3-to 6-month-olds and because of our wish to evaluate endogenous attention control when it first emerges (Colombo & Cheatham, 2006; Del Bianco et al., 2019; D'Entremont et al., 1997). The attention following task may be more optimal for 3-to 4-month-olds. The attention following task might also be improved by counterbalancing all turn orders to left versus right to ensure infant turns are not due to perseveration.

By comparing attention in controlled and naturalistic settings, our study contributes to the ongoing evaluation of the validity and generalizability of experimental measures of attention. All our analyses relied on video recordings from multiple cameras capturing third person perspectives of infants and caregivers. Head-mounted cameras and eye tracking devices will be especially useful for continued advances in our scientific understanding of attention in both controlled and naturalistic settings as well as how specific contexts influence attention (Franchak et al., 2018). For example, evidence from term infants indicates that social interactions and social experiences influence the development of sustained attention (Niedzwiecka et al., 2018; Yu & Smith, 2016).

### 4.4 Conclusions

Our study evaluated flexible attention control across multiple settings in healthy 5-month-olds born preterm or term. Cross-task stability was higher in infants with greater GA. We conclude that the consolidation of attention control increases with maturity. Our results have important implications because flexible attention control supports sharing of attention with social partners, which in turn, supports learning.

## Supplementary Material

Supplementary Information

## Figures and Tables

**Table 1:** Sample characteristics by birth status including comparisons between preterm and term infants.

	Preterm *(n =* 32)			Term *(n =* 39)			Comparisons	
	*n*	*%*		*n*	*%*		*χ*^2^(*df*)	*p*
Female infants	13	40.6		17	43.5		0.06 (1)	0.80
Male infants	19	59.4		22	56.5			
Maternal university education	22	68.8		32	82.1		1.71 (1)	0.19
	***M***	**SD**		***M***	**SD**		***t*** **(*df*)**	***p***
Gestational age at birth (weeks)	34.16	1.75		39.88	1.36		15.51 (69)	<0.001
Birth weight (grams)^a^	2066	448		3357	524		10.62 (65)	<0.001
Chronological age at testing (days)	151.84	6.11		152.44	5.83		0.42 (69)	0.34

Abbreviations: *df*, degrees of freedom; *M*, mean; *n*, number; SD, standard deviation.

a Information on birth weight was missing for four infants.

**Table 2:** Means and standard deviations of the proportions of response categories by birth status.

	Preterm *(n =* 32)			Term *(n =* 39)			Total sample (*N* = 71)	
	*M*	SD		*M*	SD		*M*	SD
Attention Following	0.48	0.34		0.42	0.31		0.45	0.32
Incorrect Turns	0.15	0.22		0.21	0.22		0.18	0.22
Look Away	0.07	0.14		0.20	0.27		0.14	0.23
No Turn	0.30	0.32		0.18	0.27		0.23	0.30

Abbreviations: *M*, mean; *n*, number in group; *N*, number in whole sample; SD, standard deviation.

**Table 3:** Results and parameters of regressions of variables from the naturalistic observations on gestational age and proportions of checking back trials in the experimental task.

Outcome: Attention to objects	Coeff.	Robust SE	*t*	*p*	*β*
Gestational age	0.08	0.04	2.07	0.043*	0.26
Proportion checking back	0.69	0.34	2.02	0.047*	0.19
Constant	−0.37	0.14	−2.65	0.010*	-
**Outcome: Responding to attention**	**Coeff.**	**Robust SE**	***t***	***p***	***β***
Gestational age	0.09	0.03	3.43	0.001**	0.39
Proportion checking back	−0.14	0.33	2.02	0.674	−0.05
Constant	−0.26	0.12	−2.65	0.030*	-

*Note:* First Model: *F*(2,68) = 4.42; *p* = 0.016; Root MSE = 0.924. Second Model: *F*(2,68) = 5.90; *p* = 0.004; Root MSE = 0.717.

* *p* < 0.05

** *p* < 0.01.

**Table 4:** Moderation model parameters.

	Coeff.	Robust SE	*t*	*p*	*β*
Gestational age	0.02	0.04	0.58	0.56	0.08
Checking Back	0.70	0.35	2.00	0.050	0.19
GA × Checking Back	0.20	0.12	1.70	0.093	0.24
Constant	−0.37	0.14	−2.69	0.009	-
