# Supplementary material for: Attention control in preterm and term 5-month-old infants: Cross-task stability increases with gestational age
Source: Infancy. Author manuscript; Available in PMC 2026 Nov 14. (PMC7619732; doi:10.1111/infa.12574)
Supplement: Supplementary file 1 [file EMS222244-supplement-Supplementary_Information.pdf]

# Supplementary Information

## S1: Preliminary Checks of Contextual Factors Effects on Infant Attention Following

In preliminary analyses of Attention Following we checked the effect of variations in factors within trials: the distance between experimenter and infant, and whether mothers supported the infant's head. To this aim we ran separate multilevel logistic regressions considering the main outcomes of the task separately: Attention Following, Checking Back, and No Turns. Each valid trial completed by an infant was categorised into dichotomous variables that indicated whether the infant displayed the response considered or not (e.g., Attention Following vs. other). The multilevel logistic regressions tested the association between the likelihood of these outcomes within each trial and the contextual factors, while allowing for the clustering of the trials at the infant level and the fact that infants could contribute different numbers of valid trials (between 1 and 4).

Logistic regressions provide log-odds parameters that can be converted into Odds Ratios, which represent the change in the odds of a response (e.g., a No Turn trial) associated with a covariate (e.g., distance between experimenter and infant above 40 cm). An accessible but detailed introduction to multilevel models applied to categorical and count data can be find in Rabe-Hesketh and Skrondal (2022). The number of participants in the analyses was 71, and the total number of valid trials was 228 (average 3.2 trials per infant, range 1 to 4). These analyses were run using the "melogit" command in Stata 17.0 (StataCorp., 2021).

Overall, the results reported in the Tables overleaf indicated there were no significant associations between Attention Following trials and trial-specific contextual factors. The results concerning associations between Checking Back and contextual factors are not reported, but they indicated no significant associations. Furthermore, the results did not indicate significant associations between likelihood of No trials and trial-specific contextual factors.

***References***

Rabe-Hesketh, S. & Skrondal, A. (2022). *Multilevel and Longitudinal Modeling Using Stata.Volume II: Categorical Responses, Counts, and Survival (4th Edition)*. College Station TX (US): Stata Press.

StataCorp. (2021). *Stata Statistical Software: Release 17*. College Station, TX: StataCorp LLC.

**Table S1.1**

*Multilevel Regression of Infants' Attention Following on Distance Experimenter-Infant*

| | *OR* | *95% CI LB* | *95% CI UB* | *z (p value)* |
|---|---|---|---|---|
| Distance > 40 cm | 0.62 | 0.30 | 1.29 | -1.27 (.20) |
| Constant | 1.08 | 0.58 | 2.00 | 0.25 (.80) |
| | *Variance* | *95% CI LB* | *95% CI UB* | |
| Within-participants | 0.45 | 0.08 | 2.51 | |

*Note.* Wald $\chi^2$ (1) = 1.62; $p$ = .20. CI LB = Confidence interval- Lower Bound; CI UP= Confidence Interval Upper Bound.

**Table S1.2**

*Multilevel Regression of Infants' Attention Following on Infants' Head being Supported*

| | *OR* | *95% CI LB* | *95% CI UB* | *z (p value)* |
|---|---|---|---|---|
| Head Supported | 0.74 | 0.32 | 1.72 | -0.69 (.49) |
| Constant | 0.81 | 0.57 | 1.56 | -1.16 (.25) |
| | *Variance* | *95% CI LB* | *95% CI UB* | |
| Within-participants | 0.48 | 0.09 | 2.53 | |

*Note.* Wald $\chi^2$ (1) = 0.48; $p$ = .49 CI LB = Confidence interval- Lower Bound; CI UP= Confidence Interval Upper Bound.

**Table S1.3**

*Multilevel Regression of Infants' No Turns on Distance Experimenter-Infant*

| | ***OR*** | ***95% CI LB*** | ***95% CI UB*** | ***z (p value)*** |
|---|---|---|---|---|
| Distance > 40 cm | 2.52 | 0.76 | 8.39 | 1.50 (.13) |
| Constant | 0.10 | 0.03 | 0.33 | -3.81 (< .001) |
| | ***Variance*** | ***95% CI LB*** | ***95% CI UB*** | |
| Within-participants | 1.77 | 0.62 | 5.05 | |

*Note*. Wald $\chi^2$ (1) = 2.26; $p$ = .13. CI LB = Confidence interval- Lower Bound; CI UP= Confidence Interval Upper Bound.

**Table S1.4**

*Multilevel Regression of Infants' No Turns on Infants' Head being Supported*

| | ***OR*** | ***95% CI LB*** | ***95% CI UB*** | ***z (p value)*** |
|---|---|---|---|---|
| Head Supported | 2.30 | 0.73 | 7.21 | 1.43 (.15) |
| Constant | 0.19 | 0.10 | 0.34 | -5.41 (<.001) |
| | ***Variance*** | ***95% CI LB*** | ***95% CI UB*** | |
| Within-participants | 1.54 | 0.50 | 4.70 | |

*Note.* Wald $\chi^2$ (1) = 2.04; $p$ = .15. CI LB = Confidence interval- Lower Bound; CI UP= Confidence Interval Upper Bound.

**S2: Ordinal Logistic Regressions of Attention Following Outcomes on Gestational Age**

For each infant we calculated the proportions of trials categorised as Attention Following, Checking Back and No Turn using the number of valid trials as the denominator. Since these proportions were calculated on a limited number of trials (3.2 per infant, on average), these outcomes had a limited number of possible responses and were not normally distributed.

To take this issue into account, the proportion of trials in each of these categories was considered an ordered categorical response. We then used ordinal logistic regressions to test the association between the outcome and gestational age, as well as other covariates, i.e., infant's sex and maternal education.

Ordinal logistic regressions are based on assessments of the cumulative response probabilities of an ordered variable. The regression analyses represent the probability that the value of an ordered variable (e.g., proportions of No Turns) is equal to one of the ordered categories (e.g., category 2) or a higher one (e.g., category 3). Using a logit-link function, the ordinal logistic regression models estimate *thresholds*, or "cut-points", which represent the log-odds of being in a category of the outcome or a higher one (e.g., category 2 or category 3), rather than being in a lower category (e.g., category 1), see Leckie and colleagues (2016) for more examples. Thresholds in the model can be considered analogous to intercepts of a standard regression model (Steel, 2011).

This parameterization allows to calculate coefficients that represent the log-odds of changes in the outcome as a function of covariates. For example, in regressing the proportion of No Turns on gestational age, we obtain an estimate of how the log-odds of being in a higher category of No Turn proportions change with every week of gestational age. A single log-odds coefficient is estimated because the model assumes that the effect of the covariate is the same whether we compared the category 1 of the outcome versus other categories, or whether we compared categories 1 and 2 versus other categories, and so on. This assumption is referred to as the *proportional odds* assumption. This assumption was tested on all three outcomes, with results indicating the assumption was tenable (more details are available under request). A general and accessible introduction to ordinal logistic models is provided by Steel (2011).

We ran these analyses using the "ologit" command in Stata 17.0 (StataCorp., 2021). We tested different ordinal logistic regression models where we compared:

(1) A linear effect of gestational age on the outcome.

(2) A quadratic effect of gestational age on the outcome.

Once we had selected the optimal model representing the association between gestational age and the outcome, we also included covariates that represented infants' sex and their mothers' educational attainment. Parameters from these models are reported in the following tables.

**Table S2.1**

*Ordinal logistic regression of Attention Following on gestational age (linear association).*

| | *Coefficient* | *95% CI LB* | *95% CI UB* | *z (p value)* |
|---|---|---|---|---|
| GA (centred) | -0.04 | -0.17 | 0.08 | -0.65 (.52) |
| Threshold 1 | -1.42 | -2.01 | -0.83 | --- |
| Threshold 2 | -0.51 | -0.99 | -0.02 | --- |
| Threshold 3 | -0.16 | -0.63 | 0.31 | --- |
| Threshold 4 | 0.85 | 0.34 | 1.36 | --- |
| Threshold 5 | 1.06 | 0.53 | 1.60 | --- |
| Threshold 6 | 1.80 | 1.13 | 2.47 | --- |

*Note*. $\chi^2$ (1) = 0.42; $p$ = .52; *Pseudo* $R^2$ = .002. GA= Gestational Age; CI LB = Confidence interval- Lower Bound; CI UP= Confidence Interval Upper Bound.

**Table S2.2**

*Ordinal logistic regression of Attention Following on gestational age (quadratic association).*

| | *Coefficient* | *95% CI LB* | *95% CI UB* | *z (p value)* |
|---|---|---|---|---|
| GA (centred) | *-0.03* | *-0.16* | *0.09* | *-0.50 (.62)* |
| $GA^2$ | *0.03* | *-0.01* | *0.08* | *1.42 (.16)* |
| Threshold 1 | *-1.08* | *-1.83* | *-0.33* | --- |
| Threshold 2 | *-0.15* | *-0.84* | *0.54* | --- |
| Threshold 3 | *0.20* | *-0.48* | *0.88* | --- |
| Threshold 4 | *1.22* | *0.49* | *1.94* | --- |
| Threshold 5 | *1.43* | *0.68* | *2.18* | --- |
| Threshold 6 | *2.18* | *1.32* | *3.04* | --- |

*Note*. $\chi^2$ (2) = 2.48; $p$ = .29; *Pseudo* $R^2$ = .001. GA= Gestational Age; CI LB = Confidence interval- Lower Bound; CI UP= Confidence Interval Upper Bound.

**Table S2.3**

*Ordinal logistic regression of Attention Following on gestational age (linear association), infants' sex, and maternal education.*

| | *Coefficient* | *95% CI LB* | *95% CI UB* | *z (p value)* |
|---|---|---|---|---|
| GA (centred) | -0.04 | -0.17 | 0.08 | -0.67 (.51) |
| Infant female | 0.09 | -0.79 | 0.97 | 0.19 (.85) |
| Maternal Univ.Degree | 0.07 | -0.98 | 1.12 | 0.13 (.90) |
| Threshold 1 | -1.34 | -2.44 | -0.24 | --- |
| Threshold 2 | -0.42 | -1.49 | 0.64 | --- |
| Threshold 3 | -0.07 | -1.14 | 0.99 | --- |
| Threshold 4 | 0.94 | -0.15 | 2.03 | --- |
| Threshold 5 | 1.15 | 0.05 | 2.25 | --- |
| Threshold 6 | 1.88 | 0.71 | 3.05 | --- |

*Note*. $\chi^2$ (3) = 0.46; *p* = 93; *Pseudo* $R^2$ = .002. GA= Gestational Age; CI LB = Confidence interval- Lower Bound; CI UP= Confidence Interval Upper Bound.

**Table S2.4**

*Ordinal logistic regression of Checking Back on gestational age (linear association).*

| | *Coefficient* | *95% CI LB* | *95% CI UB* | *z (p value)* |
|---|---|---|---|---|
| GA (centred) | 0.02 | -0.11 | 0.15 | 0.28 (.78) |
| Threshold 1 | -0.48 | -0.96 | 0.002 | --- |
| Threshold 2 | 0.44 | -0.04 | 0.92 | --- |
| Threshold 3 | 0.88 | 0.36 | 1.39 | --- |
| Threshold 4 | 2.07 | 1.33 | 2.81 | --- |
| Threshold 5 | 2.82 | 1.81 | 3.83 | --- |
| Threshold 6 | 3.55 | 2.14 | 4.95 | --- |

*Note.* $\chi^2$ (1) = 0.08; *p* = .78; *Pseudo* $R^2$ = .0003. GA= Gestational Age; CI LB = Confidence interval- Lower Bound; CI UP= Confidence Interval Upper Bound.

**Table S2.5**

*Ordinal logistic regression of Checking Back on gestational age (quadratic association).*

| | *Coefficient* | *95% CI LB* | *95% CI UB* | *z (p value)* |
|---|---|---|---|---|
| GA (centred) | 0.02 | -0.11 | 0.15 | 0.26 (.79) |
| $GA^2$ | -0.01 | -0.05 | 0.04 | -0.25 (.81) |
| Threshold 1 | -0.54 | -1.24 | 0.15 | --- |
| Threshold 2 | 0.38 | -0.31 | 1.07 | --- |
| Threshold 3 | 0.52 | 0.10 | 1.53 | --- |
| Threshold 4 | 2.01 | 1.12 | 2.89 | --- |
| Threshold 5 | 2.76 | 1.64 | 3.88 | --- |
| Threshold 6 | 3.49 | 2.00 | 4.97 | --- |

*Note*. $\chi^2$ (2) = 0.14; *p* = .93; *Pseudo* $R^2$ = .0006. GA= Gestational Age; CI LB = Confidence interval- Lower Bound; CI UP= Confidence Interval Upper Bound.

**Table S2.6**

*Ordinal logistic regression of Checking Back on gestational age (linear association), infants' sex, and maternal education.*

| | *Coefficient* | *95% CI LB* | *95% CI UB* | *z (p value)* |
|---|---|---|---|---|
| GA (centred) | 0.02 | -0.11 | 0.15 | 0.25 (.80) |
| Infant female | -0.26 | -1.15 | 0.62 | -0.58 (.56) |
| Maternal Univ.Degree | 0.26 | -0.76 | 1.27 | 0.50 (.62) |
| Threshold 1 | -0.40 | -1.44 | 0.63 | --- |
| Threshold 2 | 0.53 | -0.51 | 1.56 | --- |
| Threshold 3 | 0.97 | -0.09 | 2.03 | --- |
| Threshold 4 | 2.17 | 0.97 | 3.36 | --- |
| Threshold 5 | 2.92 | 1.54 | 4.30 | --- |
| Threshold 6 | 3.65 | 1.96 | 5.34 | --- |

*Note.* $\chi^2$ (3) = 0.87; $p$ = .83; *Pseudo* $R^2$ = .004. GA= Gestational Age; CI LB = Confidence interval- Lower Bound; CI UP= Confidence Interval Upper Bound.

**Table S2.7**

*Ordinal logistic regression of No Turns on gestational age (linear association).*

| | *Coefficient* | *95% CI LB* | *95% CI UB* | *z (p value)* |
|---|---|---|---|---|
| GA (centred) | -0.13 | -0.26 | 0.01 | -1.87 (.061) |
| Threshold 1 | 0.06 | -0.41 | 0.53 | --- |
| Threshold 2 | 0.80 | 0.29 | 1.32 | --- |
| Threshold 3 | 0.94 | 0.42 | 1.47 | --- |
| Threshold 4 | 1.83 | 1.15 | 2.50 | --- |
| Threshold 5 | 2.24 | 1.45 | 3.02 | --- |
| Threshold 6 | 3.15 | 1.99 | 4.31 | --- |

*Note.* $\chi^2$ (1) = 3.60; $p$ = .056; *Pseudo* $R^2$ = .017. GA= Gestational Age; CI LB = Confidence interval- Lower Bound; CI UP= Confidence Interval Upper Bound.

**Table S2.8**

*Ordinal logistic regression of No Turns on gestational age (quadratic association).*

| | *Coefficient* | *95% CI LB* | *95% CI UB* | *z (p value)* |
|---|---|---|---|---|
| GA (centred) | -0.18 | -0.33 | -0.03 | -2.32 (.020)* |
| $GA^2$ | -0.05 | -0.10 | -0.01 | -2.18 (.029)* |
| Threshold 1 | -0.54 | -1.25 | 0.17 | --- |
| Threshold 2 | 0.24 | -0.46 | 0.95 | --- |
| Threshold 3 | 0.39 | -0.32 | 1.10 | --- |
| Threshold 4 | 1.32 | 0.52 | 2.12 | --- |
| Threshold 5 | 1.74 | 0.86 | 2.63 | --- |
| Threshold 6 | 2.68 | 1.46 | 3.91 | --- |

*Note*. Parameters at $p$ <.05 are flagged. $\chi^2$ (2) = 8.70; $p$ = .013; *Pseudo* $R^2$ = .042. GA= Gestational Age; CI LB = Confidence interval- Lower Bound; CI UP= Confidence Interval Upper Bound.

**Table S2.9**

*Ordinal logistic regression of No Turns on gestational age (quadratic association), infants' sex, and maternal education.*

| | *Coefficient* | *95% CI LB* | *95% CI UB* | *z (p value)* |
|---|---|---|---|---|
| GA (centred) | -0.17 | -0.33 | -0.02 | -2.20 (.028)* |
| $GA^2$ | -0.05 | -0.10 | -0.01 | -2.20 (.028)* |
| Infant female | -0.32 | -1.25 | 0.61 | -0.68 (.50) |
| Maternal Univ.Degree | -0.25 | -1.30 | 0.79 | -0.48 (.63) |
| Threshold 1 | -0.86 | -2.03 | 0.30 | --- |
| Threshold 2 | -0.07 | -1.23 | 1.08 | --- |
| Threshold 3 | 0.07 | -1.09 | 1.24 | --- |
| Threshold 4 | 0.99 | -0.25 | 2.23 | --- |
| Threshold 5 | 1.42 | 0.12 | 2.72 | --- |
| Threshold 6 | 2.36 | 0.82 | 3.89 | --- |

*Note.* Parameters at $p$ <.05 are flagged. $\chi^2(4) = 9.34$; $p = .053$; *Pseudo* $R^2 = .045$. GA= Gestational Age; CI LB = Confidence interval- Lower Bound; CI UP= Confidence Interval Upper Bound.

Overall, there was no evidence of associations between Attention Following and gestational age, nor was there evidence of associations between Checking Back and gestational age. Results indicated a quadratic association between No Turns and gestational age (Table S2.8). The log-odds of higher categories of No Turns decreased with increasing gestational age, while the quadratic term indicated that this decrease was steeper with increasing gestational age. We represented the quadratic association between gestational age and proportion of No Turn trials in Figure 2 of the manuscript using estimates from a multilevel logistic regression model where No Turn trials were nested within infants (see Supplementary Information S1). We used these estimates in producing the figure because the multilevel logistic regression model provides estimates that can be plotted more clearly in a graph. The results also indicated that infants' sex and maternal education attainment were not associated significantly with the outcomes collected in the experimental task.

***References***

Leckie, G., Morris, T., Steele, F. (2016). Single-level and Multilevel Models for Ordinal Responses - Stata Practical. LEMMA VLE Module 9, 1-43. Accessed at https://www.bristol.ac.uk/cmm/learning/online-course/.

StataCorp. (2021). *Stata Statistical Software: Release 17*. College Station, TX: StataCorp LLC.

Steel, F. (2011). *Module 9: Single-level and Multilevel Models for Ordinal Responses Concepts*. LEMMA VLE, University of Bristol, Centre for Multilevel Modelling. Accessed at https://www.cmm.bris.ac.uk/lemma/.

## S3: Further Details on Regressions of Outcomes from the Naturalistic Observations on Checking Back and Gestational Age

We tested the association between the outcomes from the naturalistic observations on the proportion of Checking Back trials and gestational age (centred at 37 weeks) using multivariable regressions. We conducted the analyses using Stata 17.0 (StataCorp., 2021). However, since the proportion of Checking Back trials was not normally distributed, nor could this variable be transformed to approximate a normal distribution, we ran these regressions using the Huber/White/sandwich estimator of variance, thus estimating standard errors that were robust to misspecification. This estimation was obtained by invoking the option "robust" in the command "regress" (StataCorp., 2021).

For example, the regression of infants' proportion of Attention to Objects during the naturalistic interactions (variable: "attobj") on the proportion of Checking Back trials (variable "pCB") while controlling for centred gestational age (variable "cGA") was invoked by the command:

```
regress attobj pCB cGA, robust
```

In order to test moderation of the associations by gestational age (Aim 3), we ran a second regression whereby we included an interaction term:

```
regress attobj c.pCB##c.cGA, robust
```

The interaction term was created using Stata language whereby the symbol "##" invokes estimation of the main effects and the interaction effects of the variables at the two ends of the "##" symbol, i.e. the effects of variable pCB and variable cGA, as well as their interaction. The prefix "c." in front of the variable is used in Stata to indicate that the two variables in the interaction are considered continuous.

***Reference***

StataCorp. (2021). Stata Statistical Software: Release 17. College Station, TX: StataCorp LLC.

**S4: Experimental Attention Following Task: Multiple Imputation Methods and Results**

The analyses we report in the main manuscript were conducted on 71 participants, thus excluding 33 participants who had not completed the experimental Gaze Following task but had completed the naturalistic observations analysed in a previous paper. Among those that had not completed the experimental task, 25 (76%) were full term. In order to replicate the analyses on the full *N*=104 sample of participants from our previous study, we used Multiple Imputation (MI) to impute the missing values of the experimental task.

***Multiple Imputation Methods***

Multiple imputation is a general approach to deal with missing data. The purpose of multiple imputation is to create a set of datasets whereby the missing values in one or more variables are replaced by plausible imputed values. Statistical analyses and models are run on the imputed datasets, and the parameter estimates averaged across the *n* imputed datasets. Standard errors are calculated taking into account variability across the *n* imputed datasets, thus adequately representing uncertainty associated with the missing values.

Multiple imputation is considered a valid method for tackling missing data as long as the missing values can be plausibly assumed to be Missing Completely at Random (MCAR) or Missing at Random (MAR). MCAR describes a scenario whereby there are no systematic differences between the missing and the observed values, e.g. information from the experimental task was missing because of adverse weather that prevented parents to attend the visit. MAR describes a scenario whereby the differences between missing and observed values are associated with differences in other observed variables. For example, the information from the experimental task was missing because of infants' characteristics. A different scenario whereby Multiple Imputation would not be warranted involves data Missing not at Random (MNAR): in this case, the reason for data being missing is systematically associated with values of the missing variable itself. We assumed that the mechanisms underlying missing information in our datasets were most likely to be of the MAR type, since missing data appeared to be related with infants' gestational age.

Because our data had missing values on the different outcomes of the experimental task, we used chained equation methods to create 250 imputed datasets. Chained equations are used to fill missing data in an iterative manner. To facilitate the imputation process, in the multiple imputation we considered the proportion of Attention Following, No Turns and Checking Back trials as the outcomes of interest. Namely, we imputed the *proportions of trials* with different outcomes displayed by each participant.

The proportion of trials with different outcomes were treated as ordered categorical variables. Thus, the imputation of missing values was carried out using ordered logistic regressions where each outcome (e.g. the proportion of No Turn trials) was regressed on the proportion of other outcomes. To impute plausible missing values, gestational age was added as a covariate in the imputation process. To take into account the quadratic relationship between No Turns and gestational age, a quadratic term representing the square of gestational age was added as a covariate in the imputation of the proportion of No Turns. Attention to Objects scores from the naturalistic observations were also used as covariates in the imputation process for Attention Following and Checking Back, since these scores were related.

We conducted multiple imputation in Stata software version 17.0 (StataCorp., 2021), using the ‘mi impute chained’ command suite. After creating the 250 imputed datasets for all cases in the analyses, parameters were estimated using ‘mi estimate:’ command in Stata. These methods use Rubin’s rule to average parameter estimates across the datasets with imputed complete values.

***Multiple Imputation Results***

The results obtained running tests on the complete imputed data of the *N*=104 participants confirmed the pattern of results described in the manuscript. Firstly, both preterm and term infants reported average Attention Following scores that were significantly above the level of chance (i.e., 0.25), *mean* = 0.49 (*95% Confidence Intervals* 0.37 to 0.61) and *mean* = 0.43 (*95% Confidence Intervals* 0.33 to 0.53) for preterm and term infants respectively. Furthermore, no differences were observed between preterm and term infants in Attention Following or Checking Back measures (results obtainable under request), whereas the results of ordinal logistic regressions confirmed a quadratic trend whereby the proportion of No Turns decreased with GA, with a steeper decrease with maturation, see Table S4.1. Further analyses also confirmed that Attention Following and Checking Back were not related to GA (results not displayed, available under request).

**Table S4.1**

*Ordinal Logistic Multiple Imputation Coefficients of Proportions of No Turns on Gestational Age (GA) Squared.*

| | ***Coef.*** | ***95% CI LB*** | ***95% CI UB*** | ***t (p value)*** |
|---|---|---|---|---|
| GA | -0.16 | -0.31 | -0.003 | -2.00 (.046) |
| $GA^2$ | -0.05 | -0.10 | -0.002 | -2.03 (.042) |
| Threshold 1 | -0.61 | -1.34 | 0.12 | |
| Threshold 2 | 0.13 | -0.58 | 0.83 | |
| Threshold 3 | 0.28 | -0.42 | 0.99 | |
| Threshold 4 | 1.13 | 0.32 | 1.95 | |
| Threshold 5 | 1.55 | 0.66 | 2.43 | |
| Threshold 6 | 2.45 | 1.27 | 3.62 | |

*Note*. $N$ = 104*;* Parameters estimated across 250 imputed datasets; $F$ (2, 4908.8) = 3.60; $p$ = .027.

***Reference***

StataCorp. (2021). Stata Statistical Software: Release 17. College Station, TX: StataCorp LLC.

## S5: Cross-Task Stability: Multiple Imputation Methods and Results

### *Multiple Imputation Methods*

We used chained equation methods to impute the missing values of Checking Back of the $n$=33 who had not completed the experimental task and created 250 imputed datasets. We considered the outcomes of interest to be ordered categorical variables: the values these proportions could assume were in fact bounded by the number of test trials delivered ($n$ = 4). Because the model of interest had different conditional specifications that included product effects (e.g., interactions), we imputed missing values using the *Substantive Model Compatible Fully Conditional Specification* method. This method is implemented by the 'smcfcs' command for Stata, developed by Bartlett and Morris (2015). To improve the prediction of the outcomes of interest, ordinal logistic regressions included Attention to Objects and Gestational Age (GA) as covariates. To allow for moderation effects across and preterm and term infants, the imputations were conducted separately for these two groups. The regressions presented in the manuscript, sections *Aim B* and *Aim C* of the Results, were then replicated using the 'mi estimate:' command, which allows to compute averaged estimates across the complete imputed datasets using Rubin's rules. All the analyses were conducted in Stata 17 (StataCorp., 2021).

In order to test reliability of results concerning the ranking of infants' scores in Attention to Objects (naturalistic observations) and Checking Back (experimental task), we used the imputation method described in the previous section (Supplementary Information S4) and ranked infants' scores in Attention to Objects and Checking Back variables across the 250 imputed datasets. Ties in ranked scores were allowed for. We then used the "mi estimate" command in Stata 17 (StataCorp. 2021) to regress the absolute differences in ranks on gestational age across the 250 imputed datasets (see *Aim C* section in the Results of the manuscript).

### *Multiple Imputation Results*

The results obtained on the $N$=104 participants who provided complete or partially complete information confirmed the associations between Checking Back in the experimental task and Attention to Objects in the naturalistic task to vary with gestational age. Parameter estimates from the regression models are reported in the Tables S5.1 to S5.2. The regression that allowed gestational age to moderate the association between Checking Back and Attention to Objects (Table S5.2) indicated a significant fit of the moderation model, $F$ (3, 95.5) = 3.09, $p$ = .031.

**Table S5.1**

*Multiple Imputation Regression coefficients of Attention to Objects on Checking Back and Gestational Age*

| | ***Coef*** | ***Robust SE*** | ***t (p value)*** |
|---|---|---|---|
| Checking Back | 0.75 | 0.40 | 1.85 (.069) |
| Gestational Age | 0.08 | 0.03 | 2.29 (.024) |
| Constant | -0.17 | 0.15 | -1.12 (.27) |

*Note*. $N = 104$; Parameters estimated across 250 imputed datasets; A robust estimator of variance was used. $F\ (2, 96.8) = 4.48$; $p = .014$.

**Table S5.2**

*Multiple Imputation Regression coefficients of the Moderation Model of Attention to Objects on Checking Back and Gestational Age*

| | ***Coef*** | ***Robust SE*** | ***t (p value)*** |
|---|---|---|---|
| Checking Back (CB) | 0.66 | 0.42 | 1.58 (.12) |
| Gestational Age (GA) | 0.04 | 0.05 | 0.83 (.41) |
| CB X GA | 0.14 | 0.14 | 1.03 (.30) |
| Constant | -0.15 | 0.15 | -0.98 (.33) |

*Note*. $N = 104$; Parameters estimated across 250 imputed datasets; A robust estimator of variance was used. $F\ (3, 95.5) = 3.11$; $p = .031$. CB: Checking Back; GA: Gestational Age.

Results also confirmed a significant linear association between gestational age and the absolute difference in infants' ranks in Attention to Objects and Checking Back. When this association was estimated on the $N = 104$ with complete and incomplete data, the association between the absolute difference in ranks and gestational age was significant and negative (Table S5.3). Overall, the pattern of results confirmed the reliability of the results indicating that the consistency of performance across the naturalistic observation and the experimental task increased with gestational age.

**Table S5.3**

*Multiple Imputation Regression Coefficients of the Absolute Difference of Individual Ranks in Attention to Object and Checking Back on Gestational Age*

| | ***Coef*** | ***SE*** | ***t (p value)*** |
|---|---|---|---|
| Gestational Age | -1.72 | 0.79 | -2.19 (.031) |
| Constant | 33.58 | 2.54 | 13.21 (<.001) |

*Note*. *N* = 104; Parameters estimated across 250 imputed datasets; $F_{(1, 84.3)} = 4.79$, $p = .032$

***References***

Bartlett, J., & Morris, T. (2015). "SMCFCS: Stata module to perform multiple imputation of covariates by substantive model compatible fully conditional specification," *Statistical Software Components S457968*, Boston College Department of Economics, revised 16 Feb 2019.

StataCorp. (2021). Stata Statistical Software: Release 17. College Station, TX: StataCorp LLC.
